# Amylase-assisted extraction alters nutritional and physicochemical properties of polysaccharides and saponins isolated from *Ganoderma* spp.

**DOI:** 10.1016/j.fochx.2023.100913

**Published:** 2023-09-29

**Authors:** Bo Jie Chen, Yang Liu, Ke Yang, Xia Li, Xinhong Dong, Yuan Guan, Amin Ismail, Hock Eng Khoo

**Affiliations:** a Guangxi Key Laboratory of Electrochemical and Magneto-chemical Functional Materials, College of Chemistry and Bioengineering, Guilin University of Technology, Guilin 541006, China; b South Asia Branch of National Engineering Research Center of Dairy Health for Maternal and Child Health, Guilin University of Technology, Guilin 541006, China; c Department of Nutrition, Faculty of Medicine and Health Sciences, Universiti Putra Malaysia, 43400 Serdang, Selangor, Malaysia

**Keywords:** Functional food, Reishi mushroom, Thermal stability, Wood fungus

## Abstract

This study aimed to evaluate the efficacy of amylase in hydrolyzing complex carbohydrates of different parts of *Ganoderma* spp. The aqueous extracts of the *Ganoderma* samples were analyzed for their selected nutritional composition and physicochemical properties. The purified extracts were also structurally characterized. The aqueous canopy extracts of red–purple *Ganoderma* had a notably higher total sugar and saponin content than their stalks, but not for the black-type *Ganoderma*. The enzymatic extraction effectively improved the extraction yields, whereas the amounts of sugars and saponins in some extracts were increased after the enzymatic treatment. The results also showed that only those enzyme-treated cultivated black *Ganoderma* canopy had increased total sugar and total saponin content. The antioxidant activities of all stalk extracts were higher than the canopy extracts. Their emulsifying properties were comparable with lecithin due to their high saponin content. Therefore, these extracts are new natural emulsifiers.

## Highlights

• A study of canopies and stalks of wild types and cultivated *Ganoderma.*
• Enzymatic extraction yielded a higher saponin content in the canopy of black-type *Ganoderma*.
• Purified *Ganoderma* extracts had molecular weights ranging from 1400 to 3250 Da.
• Emulsification activity of the *Ganoderma* samples was better than lecithin.
• *Ganoderma* saponins had good thermal stability even at a temperature of 800 °C.

## Introduction

*Ganoderma* (Ga), also known as lingzhi and reishi, is a traditional medicinal plant with many beneficial effects. It is a Chinese cultural treasure. *Ganoderma lucidum* is native to eastern Asia, especially China, where it is the most widely distributed. There are over a hundred species of Ga found worldwide, and the more common varieties in China are red (*G. lucidum*), purple (*G. sinensis*), and black (*G. atrum*) types. The color of red Ga is reddish brown, and some even look purplish. It is also known as reishi mushroom. The black-type Ga has a typical dark brown or blackish look.

The wild Ga mushroom grows in a harsh environment, lacking particular nutrients and unpredictable hostile weather, limiting its growth ability. The growing condition of the cultivated Ga is controlled and stable, enriched with enough growth nutrients, so it looks more attractive than the wild type. The fruiting bodies of the cultivated Ga tend to be larger with a brighter color than those grown in the wild. It can be grown on a large scale. The price of wild Ga is far higher than the farmed type. If the physicochemical and antioxidant properties of these wild-type and cultivated Ga are similar, the farmed Ga can be widely used as a functional food for promoting good health and preventing certain chronic diseases like cardiovascular diseases and cancers.

The wild-type black *G. atrum* has a small canopy attached to a long, tiny stalk. The cultivated black type has a higher proportion of the canopy part than its stalk. The wild-type red and purple Ga also has a smaller canopy proportion than the cultivated Ga. The difference in appearance between the farmed and wild types could be related to the growth environment and nutrients. The growing temperature, humidity, and other intrinsic factors of the Ga cultivation are relatively similar, which ensures a stable growth of the fruit bodies. Ga contains hundreds of bioactive compounds, mainly polysaccharides and triterpenes (Basnet et al., 2017). Ga triterpenes are the most promising bioactive substances for disease prevention.

The commonly used methods for extracting bioactive components from Ga samples include hot water and organic solvent extractions. Some researchers employed ultrasonic-assisted and enzymatic extractions. The extraction of polysaccharides from Ga using aqueous ethanol combined with ultrasonic-assisted extraction increased the polysaccharide extraction yield (Zheng et al., 2020). Due to the improved living standards, more people opt for functional drinks prepared from this medicinal plant, which can improve health and enhance immunity. Because of the positive effects of polysaccharides, flavonoids, and saponins extracted from Ga mushrooms on human health, a study of bioactive components in the Ga extracts may benefit the healthy population and people with diseases. This research insight may provide the basis for developing pharmaceutical ingredients using Ga polysaccharides and saponins.

To the best of our knowledge, there is a lack of evidence regarding the nutritional composition, *in vitro* antioxidant capacity, emulsification activity, and heat stability of various parts of different Ga species. As emulsifiers are widely used in many aspects of our lives, including the cosmetics and food industries, it is necessary to isolate and study a novel emulsifier from Ga samples. Therefore, we aimed to determine the nutritional composition, antioxidative, and physicochemical properties of the hydrophilic extract of wild and cultivated Ga. The novelty of this study includes the enzyme-treated Ga saponin analysis and determination of its physicochemical characteristics.

## Materials and methods

### Samples

Two wild-type and two cultivated Ga samples were obtained from a local herbal wholesale market in Guilin City. The wild-type samples were the brownish *G. applanatum* and black-type *G. hainanense*. Purplish *G. sinensis* and black *G. atrum* were the cultivated Ga. Images of these Ga samples are shown in Fig. S1. The black-type (B-type) Ga samples originated from Yunnan province, whereas the other reishi samples (R-type) were of Guangxi origin. Black (B)-type referred to the Ga mushrooms with black fruiting bodies, whereas the reishi (R)-type referred to the Ga mushrooms with purplish to brown fruiting bodies. The Ga samples were labeled as RCC (canopy sample of cultivated *G. sinensis*), RCS (stalk sample of cultivated *G. sinensis*), RWC (canopy sample of wild *G. applanatum*), RWS (stalk sample of wild *G. applanatum*), BCC (canopy sample of cultivated *G. atrum*), BCS (stalk sample of cultivated *G. atrum*), BWC (canopy sample of wild *G. hainanense*), BWS (stalk sample of wild *G. hainanense*).

### Preparation of Ga extracts

The crude lipid or fat in the oven-dried Ga samples was separated using *n*-hexane by soaking in a locally produced water bath at 60 °C for 6 h. The aqueous extracts were prepared applying ultrasonic-assisted hot water-enzymatic and non-enzymatic extractions (Baiseitova et al., 2022). The lipid in the Ga powder (100 g) was initially extracted using 500 mL of hexane and then oven-dried at 60 °C. The fat extraction was repeated twice for each sample. After the fat removal, the defatted Ga powder was subjected to enzymatic extraction of sugars and saponins. The defatted Ga powders were subjected to enzymatic extraction using amylase produced from *Aspergillus oryzae* (Rhawn Chemical Reagent Co., Ltd., Shanghai, China).

In brief, 1.0 g of defatted Ga powder was added with 50 mL distilled water containing 2 U/mL amylase. The sample was incubated at 60 °C for 6 h during the hot-water enzymatic reaction, followed by ultrasonic-assisted extraction for 30 min. The crude extracts were lyophilized using a freeze dryer (Alpha1-2LD Plus; Martin Christ GmBH, Osterode, Germany) at − 40 °C for 72 h. The extraction yields were then calculated based on the percentages of lyophilized extracts. The extraction yields of these Ga samples were subjected to a comparison between water extraction and enzymatic extraction methods. The water extraction was performed according to the same procedure for enzymatic extraction without adding the amylase during the extraction.

The flavonoids and other semi-polar compounds in the extracts were removed using methanol after the initial enzymatic extraction. The sugars and saponins in the sample residues were re-extracted using hot-water extraction with the enzymatic treatment. The aqueous extracts were then concentrated at 60 °C, centrifugated at 4,000 rpm to remove the undigested complex carbohydrate, and passed through 2.0 g-activated carbon cartridges (200-mesh carbon particle size). The Ga extracts with their color removed were finally lyophilized using a freeze dryer. The ash content of the Ga powder and the moisture content of the lyophilized Ga extract were determined using the AOAC methods (Liu, 2019). The water extracts were initially analyzed for their extraction yield, total sugar, and total saponin content. Comparisons were made between water and enzyme extracts. Based on the results obtained, only lyophilized enzyme extracts were subjected to nutritional and physicochemical analyses, except for moisture and ash determinations.

### Determination of total sugar, total saponin, and total protein content

The total sugar content (TSC) of the Ga extracts was determined using the phenol–sulfuric acid colorimetric method (Sun et al., 2021). Total saponin content of the Ga extracts was determined using a colorimetric method reported in the literature (Kurkin and Akushskaya, 2014). Total protein content of the Ga extracts was determined based on the Coomassie staining method (Sun et al., 2021).

### Analysis of monosaccharide and trehalose

The analysis of monosaccharides was performed after the monosaccharide derivatization. The Ga extracts (2 mg) were pretreated with 1 mL of 1 M hydrochloric acid for 8 h at 80 °C and then hydrolyzed using 1 mL of 2 M trifluoroacetic acid at 120 °C for 1 h. The sugar derivatization was performed using PMP (1-phenyl-3-methyl-5-pyrazolone). In brief, 0.5 mL of 0.5 M PMP and 0.5 mL of 0.3 M sodium hydroxide were added to the acid-hydrolyzed extract, thoroughly mixed, and reacted in the water bath at 70 °C for 30 min. After centrifugation, 0.05 mL of 0.3 M HCl solution and 0.05 mL ultrapure water were added to the 0.1 mL of the derivatized extract solution. The final solution was filtered using a 0.22 μm syringe filter before being injected into the injection valve of the LC-20A HPLC system (Shimadzu, Japan).

The trehalose and monosaccharide compositions of the Ga extracts were determined according to the method described in the literature with some modifications (Liu et al., 2021). The sugars in the extract solution were separated using an Inertsil ODS-3 column (4.6 × 150 mm). The injection volume was 2 μL, and the monosaccharides and trehalose were separated by applying an isocratic mobile phase consisting of phosphate buffer solution (0.1 M, pH 7.0) and acetonitrile at a ratio of 82:18. The flow rate was set at 1.0 mL/min, the total run time was 40 min, and the detection wavelength was fixed at 245 nm. HPLC grade trehalose (Tre), mannose (Man), ribose (Rib), galactose (Gal), glucose (Glu), xylose (Xyl), and arabinose (Ara) of ≥ 98 % purity were used as the sugar standards (Yuanye Biotechnology Co., Ltd., Shanghai, China).

### Determination of molecular weights

The molecular weight distribution of the Ga extracts was analyzed using a size exclusion chromatographic method (Xie et al., 2016). Ga polysaccharides of different molecular weights were separated using a TSKgel G4000PWXL column (300 mm × 7.8 mm) connected to a Prominence LC-20A Modular HPLC System (Shimadzu Corporation, Japan). The detector was a differential refractive index detector (RID-20A). A 2 mg/mL extract solution was prepared and filtered before the chromatographic analysis. The isocratic mobile phase was sodium chloride solution containing 0.02 % (*w*/*v*) sodium azide. The injection volume was 2 μL, and the flow rate was 0.6 mL/min. The total run time was 30 min, and the spectra were recorded at 254 nm. The chromatographic peaks of the sample were analyzed based on the corresponding retention times. Dextran (Yuanye Biotechnology Co., Ltd., Shanghai, China) with different molecular weights (20, 40, 70, 150, and 250 kDa) was used to plot the standard calibration graph. The molecular weights of the polysaccharide fragments retained as the chromatographic peaks were calculated using the equation (y =  − 0.2838x + 1.9091, R^2^ = 0.9792) obtained from the standard calibration curve.

### Microstructure imaging and elemental analysis

The microstructure images of the lyophilized powder of Ga extracts were captured using an SU5000 field emission scanning electron microscope (SEM, Hitachi, Tokyo, Japan), which was performed according to the method described in the literature (Xu et al., 2023). Triplicate scanning was obtained for each sample. In brief, a thin layer of Ga extract powder (2 mg) or glucose powder (2 mg) was spread on the conductive adhesive (1 cm) and uniformly covered its surface, and then platinum was sputtered on it to cover the sample surface for conductive treatment. AR grade of d-glucose powder was purchased from Xilong Science Co., Ltd. (Shantou, China). The elemental analysis of the Ga extracts was performed using the SEM-energy dispersive spectrometer (EDS). The EDS coupled with SEM at an amplification voltage of 15 kV was applied to determine the elemental composition (Jenkinson et al., 2022). The detected elements were grouped into four categories. They were the bulk elements, macrominerals, microminerals, and heavy metals.

### Fourier-transform infrared spectroscopic analysis

The Fourier transform infrared (FTIR) spectroscopic analysis was performed using the method described in the literature (Sun et al., 2021). The FTIR analysis was performed using a Nicolet iS10 FTIR spectrometer (Thermo Scientific, Waltham, USA). The spectrum scanning ranged between 4000 cm^−1^ and 400 cm^−1^. About 1 mg of the Ga extract was added to 50 mg of potassium bromide powder, mixed and pulverized. Each sample was scanned three times and plotted as a triplicate combined spectrum. The peaks corresponding to specific functional groups were identified based on the FTIR spectrum information and bond relationship reported in the literature (Xu et al., 2013, Zhang, 2023).

### Thermal gravimetric analysis

The thermal properties of the Ga extracts were determined using an STD Q600 thermogravimetric analyzer (TA Instruments, USA). The analysis was performed by referring to the procedure described in the literature with some modifications (Mohan et al., 2018). Approximately 10 mg Ga extract was obtained and then transferred to an alumina crucible and heated from 30 °C to 800 °C at a heating rate of 10 °C/min, with a nitrogen flow rate of 100 mL/min.

### Emulsion separation index

The emulsification activity of the Ga extracts was determined based on the emulsion separation index (Shen et al., 2020). Different amounts (15, 30, 45, 60, and 75 mg) of the Ga extract were dissolved in an aqueous solution containing 0.05 % sodium azide at a final volume of 7.5 mL and then added with 2.5 mL of glycerol trioleate. The mixture was homogenized using a high-speed homogenizer at 10,000 rpm for 5 min. The upper phase was labeled the emulsion layer, whereas the lower phase was the aqueous layer. Distilled water was used as a control, and lecithin extracted from soybean (>90 %) was used for comparison. The emulsified samples were capped and sealed and finally stored at room temperature for eight days. The emulsification effect of the Ga extracts was calculated using Eq. (1) and expressed as an emulsion separation index (ESI).

(1) $$\mathrm{ESI}\;\left(\%\right)\;=\;\left(H_{S}/H_{E}\right)\times 100$$

*H_S_* is the height of the water layer, and *H_E_* is the total height of the emulsion.

### In vitro antioxidant activities

The DPPH radical scavenging activity assay of the Ga extracts was performed according to the method reported previously with slight modification (Farooq et al., 2023). In brief, DPPH reagent (0.04 mg/mL) was prepared by dissolving it in anhydrous ethanol. A 2.0 mL of 0.04 mg/mL DPPH reagent was mixed with 2.0 mL of extract solution (0, 2, 5, 10, 15, 20, 25, and 30 mg/mL). The mixture was agitated and placed in the water bath with a temperature set at 25 °C for 30 min. The absorbance was measured at 517 nm. Distilled water was used as a blank solution to replace the Ga extract. The DPPH radical scavenging activity of the Ga extracts was calculated using the equation as follows:

(2) $$\mathrm{DPPH}\;\mathrm{radical}\;\mathrm{scavenging}\;\mathrm{activity}\;\left(\%\right)\;=\;\left[1-\left(A_{2}-A_{1}\right)/A_{0}\right]\times 100$$

*A_0_* is the absorbance of the blank, *A_1_* is the absorbance of the control, and *A_2_* is the absorbance of the sample. EC_50_ values were calculated using the equation obtained from the linear curve plotted according to these extract concentrations.

The reducing power of the Ga extracts was evaluated using the previously reported method (Mota et al., 2023). It was also known as ferric-reducing antioxidant power (FRAP). A 0.5 mL of Ga extract was mixed with 0.5 mL of 1 % potassium ferricyanide solution and 0.5 mL of phosphate buffer (0.2 M, pH 6.7) and then placed in a water bath (50 °C) for 20 min. After cooling to room temperature, 0.5 mL of 10 % trichloroacetic acid solution (TCA) was pipetted to the mixture. After centrifugation, the supernatant was collected and mixed with 0.5 mL of 0.1 % ferric chloride solution and 2.0 mL of distilled water and left for 10 min at the room temperature of 25 °C. The absorbance was measured at 700 nm. A standard curve was plotted using ferrous sulfate at five concentrations ranging between 0 and 6.58 μM. The FRAP values were expressed as μM Fe^2+^/g extract.

### Statistical analysis

All data were expressed as mean ± standard error (SE). The results were statistically analyzed using SPSS version 26.0 (SPSS Inc., Chicago, IL, USA). Analysis of variance coupled with the Tukey range test was used to compare the mean differences among different groups, and *P* < 0.05 was considered statistically significant.

## Results and discussion

### Extraction yields

The extraction yields of all Ga samples are shown in Table 1. The results showed that the extraction yields of all canopy samples were significantly higher than those of stalk samples (*P* < 0.05). In contrast, no significant differences in the extraction yields were observed between the canopy or stalk samples of B-type and other reishi samples (*P* > 0.05). The enzymatic extraction coupled with the high-temperature ultrasonication had significantly increased extraction yields of all Ga samples at *P* < 0.05. The water-extracted RWC had the highest extraction yield among all water-extracted Ga samples; the enzymatic-assisted extraction yield for the BCC sample was the highest among the enzymatic-extracted samples. On the contrary, the enzyme-extracted BCS had the highest increment in extraction yield.

**Table 1:** Extraction yields, total sugar, and total saponin content of different Ga samples.

**Parameters**	**RCC**		**RCS**		**RWC**		**RWS**	
	**Water extract**	**Enzymatic extract**	**Water extract**	**Enzymatic extract**	**Water extract**	**Enzymatic extract**	**Water extract**	**Enzymatic extract**
**Extraction yield (%)**	2.62 ± 0.10^b^	8.14 ± 0.16*^,a^	1.52 ± 0.06^d^	5.76 ± 0.13*^,d,e^	2.93 ± 0.04^a^	8.40 ± 0.15*^,a^	1.54 ± 0.18^d^	5.47 ± 0.20*^,e^
**Total sugar** **(mg/g)**	131.17 ± 0.14^c^	30.46 ± 1.73*^,c^	97.80 ± 1.16^d^	3.84 ± 0.04*^,d^	246.59 ± 2.56^a^	78.02 ± 0.63*^,b^	189.37 ± 5.62^b^	1.94 ± 0.03*^,d^
**Total saponin** **(mg/g)**	66.03 ± 1.90^a^	51.34 ± 0.04*^,b^	52.29 ± 0.20^c^	46.17 ± 0.03*^,e^	53.32 ± 0.34^b^	51.97 ± 0.08*^,c^	52.84 ± 0.37^c^	46.44 ± 0.11*^,e^
	**BCC**		**BCS**		**BWC**		**BWS**	
	**Water extract**	**Enzymatic extract**	**Water extract**	**Enzymatic extract**	**Water extract**	**Enzymatic extract**	**Water extract**	**Enzymatic extract**
**Extraction yield (%)**	2.19 ± 0.07^c^	8.48 ± 0.16*^,a^	1.44 ± 0.06^d^	6.21 ± 0.17*^,c,d^	2.69 ± 0.09^a,b^	7.16 ± 0.18*^,b^	1.93 ± 0.07^c^	6.29 ± 0.15*^,c^
**Total sugar** **(mg/g)**	68.39 ± 1.81^e^	240.16 ± 1.01*^,a^	28.02 ± 0.33^f,g^	3.75 ± 0.01*^,d^	21.10 ± 1.42^g^	2.86 ± 0.04*^,d^	29.62 ± 0.20^f^	1.71 ± 0.03*^,d^
**Total saponin** **(mg/g)**	52.20 ± 0.27^d^	98.90 ± 0.38*^,a^	50.60 ± 1.11^c^	47.37 ± 0.39*^,d^	46.50 ± 0.11^c^	47.29 ± 0.09^d^	45.99 ± 0.11^c^	46.47 ± 0.06*^,e^

The results obtained from this study showed that the canopy samples of both R-type and B-type Ga had notably higher extraction yields than their stalks. It indicated that the amount of water-soluble polysaccharide in the Ga canopy was higher than that of its stalk. Also, these Ga polysaccharides consisted of linear β(1–3)-glucan and branched β(1–6)-glucan (Baby et al., 2015). Therefore, adding amylase during the sonication-assisted hot water extraction could also increase the extraction rate of the Ga stalk samples besides their canopies.

The cell wall of an edible fungus is composed of chitin and glucan. These components are polysaccharides. Most of these bioactive components are found in the cytoplasm of the fungus. The ultrasonic-assisted aqueous extraction method effectively isolated these polar bioactive components or water-soluble polysaccharides from the cell walls of these Ga samples. The different polysaccharide chains intertwine to form robust chains. Amylase can untangle these intertwined-long chains to a certain extent, thus causing local collapse, softening, and loosening of the dense cell wall.

The variation in the extraction yields of different Ga samples could be related to their growing environment. The growing conditions of the cultivated Ga mushrooms are mainly prolonged hours of exposure to light, controlled temperature, and growth factors like complete plant nutrition. The growing of Ga mushrooms in the wild is subjected to climate changes and the availability of nutrients from the surroundings. When more carbohydrates are stored in the interstitium of the mushrooms, the damaged cell receptors in the interstitium are repaired in time. As a result, the extraction yields of these wild-type Ga samples were higher than those from the cultivated samples.

### Proximate composition

#### Total sugar content

TSC of enzyme and non-enzyme-treated Ga extracts is presented in Table 1. The results showed that the R-type Ga extracts had a notably higher TSC than the B-type Ga extracts, except for the enzyme-treated stalk extracts. The canopy extracts also had a higher TSC than the stalk extracts, except for the water-extracted B-type wild Ga. The enzymolysis reduced the TSC in all Ga extracts except for the BCC extract. The TSC in all Ga extracts differed significantly between the enzymatic and non-enzyme-treated samples (*P* < 0.05). Only enzyme-treated BCC (240.16 mg/g) had a 2.5 times higher TSC than the non-enzyme-treated BCC (68.38 mg/g). Therefore, the ultrasonic-assisted enzymolysis effectively increased the TSC of BCC.

The determination of TSC was performed using the phenol–sulfuric acid colorimetric method. During the reaction with sulfuric acid, the monosaccharides collected from the enzyme-assisted hot water extraction are decomposed by the acid. The enzyme-extracted Ga sample had these polysaccharides digested by amylase during extraction. Therefore, this method could give a higher total sugar value to non-enzyme-treated Ga extracts. It is a possible explanation for the higher amounts of total sugars in the canopy of non-enzyme-treated Ga extracts.

On the other hand, polysaccharides are sugar polymers with molecular structures containing glycosidic linkages. Sulfuric acid is less effective at hydrolyzing polysaccharides into reducing sugars. Acid hydrolysis is also less effective in breaking down the glycosidic bond of the polysaccharides than the enzymatic reaction. If the Ga extract contains higher numbers of aldehydes, the polysaccharides in the Ga extract will be more difficult to achieve complete hydrolysis. The release of most neutral sugars is depended on the hydrolysis temperature, time, and acid concentration (Wang et al., 2016).

#### Total saponin content

The total saponin content of the enzyme and non-enzyme-treated Ga extracts is shown in Table 1. The results showed that the total saponin content of hot-water-extracted canopy extracts of R-type Ga was higher than the B-type Ga, where RCC extract had the significantly highest total saponin content (66.03 mg/g). Similarly, the enzyme-treated R-type Ga extracts had a higher total saponin content than the enzyme-treated B-type Ga extracts, except for the stalk extracts of wild Ga. Two enzyme-extracted Ga samples (RCS and BWC) had a slight decrease in total saponin content. The results also indicated that the total saponin content of the canopy extracts was notably higher than their stalk extracts. Moreover, enzymolysis increased the saponin content of BCC extract, besides its higher extraction yield and TSC.

The high total saponin content of the BCC extract could be due to the higher extraction yield after the enzymatic treatment. The improved extraction method could yield a higher saponin content in the Ga sample. Since amylase is highly specific for cleaving glycosidic bonds of the polysaccharide, the enzyme can help release bound saponin molecules attached to the polysaccharide structure. An increased rate of saponin extraction from the saponin-rich plant samples is essential because it can reduce saponin degradation. Also, most saponins can lower the surface tension of water and act as emulsifiers. Emulsifiers play a crucial role in the formulation of many biochemical systems. Besides reducing interfacial tension, an emulsifier enhances further droplet destruction, provides a protective layer around the droplet, improves its long-term stability, and inhibits droplet aggregation. Moreover, the saponin-rich extract of BCC obtained based on the enzymolytic method is a novel pharmaceutical ingredient or an emulsifier.

#### Total fat and total protein content

The total fat content (TFC) of the Ga samples is presented in Table 2. The TFC of R-type Ga samples was significantly higher than that of B-type Ga samples (*P* < 0.05). The cultivated Ga samples also had higher TFC than the wild-type samples. Moreover, the TFC of all canopy samples of Ga was significantly higher than their stalk samples, except for the cultivated black Ga.

**Table 2:** Proximate compositions and antioxidant activities of enzymatic extracts of different Ga samples.

**Parameters**	**RCC**	**RCS**	**RWC**	**RWS**	**BCC**	**BCS**	**BWC**	**BWS**
***Proximate composition***								
**^#^Total fat (%)**	1.09 ± 0.05^a^	0.96 ± 0.02^b^	0.85 ± 0.06^c^	0.56 ± 0.03^d^	0.92 ± 0.01^b,c^	0.84 ± 0.04^c^	0.62 ± 0.01^d^	0.45 ± 0.01^e^
**Total protein (μg/g)**	2.48 ± 0.15^c^	2.33 ± 0.08^c,d^	3.36 ± 0.11^a^	3.65 ± 0.09^a^	3.47 ± 0.08^a^	2.99 ± 0.17^b^	2.05 ± 0.07^d^	2.60 ± 0.12^c^
**Total moisture (%)**	16.77 ± 0.89^b,c,d^	9.95 ± 1.44^f,g^	13.91 ± 0.42^c,e^	7.93 ± 0.20^f,h^	17.83 ± 2.33^a,b^	14.11 ± 0.41^d,e^	21.14 ± 0.63^a^	6.38 ± 1.52^g,h^
**^#^Total ash (%)**	1.14 ± 0.05^f^	0.81 ± 0.09^g^	2.82 ± 0.06^c^	9.18 ± 0.01^a^	1.35 ± 0.02^e^	1.43 ± 0.01^e^	2.49 ± 0.01^d^	3.10 ± 0.02^b^
*Saccharide ratio	0.93:0:0.07:0:0:0	0.93:0:0.07:0:0:0	0.53:0.05:0.14:0.15:0.09:0.04	0.97:0:0.03:0:0:0	0.64:0.02:0.34:0:0:0	0.94:0:0.06:0:0:0	0.96:0:0.04:0:0:0	0.97:0:0.03:0:0:0
**Molecular weight (Da)**	3,238.79; 1,413.83	3,263.75; 1,403.02	3,220.20; 1,430.20	3,260.62; 1,398.99	3,057.63; 1,520.77	3,229.49; 1,396.31	3,251.25; 1,393.63	3,260.62; 1,393.63
***Antioxidant activities***								
**DPPH** **(EC_50,_ mg/mL)**	14.66 ± 0.79^b,c^	13.25 ± 0.84^c^	11.63 ± 0.28^d^	10.91 ± 0.19^d^	18.72 ± 0.17^a^	14.50 ± 0.21^b,c^	18.99 ± 0.27^a^	15.75 ± 0.58^b^
**FRAP** **(μM Fe^2+^/g)**	0.07 ± 0.001^b^	0.08 ± 0.002^a^	0.06 ± 0.001^c^	0.08 ± 0.001^a^	0.07 ± 0.002^b,c^	0.08 ± 0.002^a^	0.02 ± 0.002^d^	0.02 ± 0.002^d^

*Monosaccharide ratio of Tre, Man, Glu, Gal, Xyl, and Ara. **^#^** denotes the total content for the oven-dried different Ga samples.

As reported in the literature, the TFC of different Ga species ranged between 1 % and 5 % (Fraile-Fabero et al., 2021). The TFC of RCC was higher than 1 %, whereas it was determined to be lower in the other Ga samples. The high fat content in the cultivated Ga samples could be because they were grown using the sterilized lignocellulosic biomass at a controlled temperature and had better nourishment than the wild-type samples. Some of these biomasses have been formulated by adding 1 % sugar and 1 % calcium sulfate. Mineral-enriched biomass could have affected lipid accumulation in the mycelium of the cultivated Ga mushrooms.

The total protein content (TPC) of the enzyme-treated Ga extracts was estimated using the Coomassie staining method, and the results are shown in Table 2. The wild R-type Ga extracts had a significantly higher TPC than the cultivated R-type Ga extracts (*P* < 0.05), but the TPC of the B-type cultivated Ga extracts was higher than the wild B-type Ga extracts. There are several explanations for the detection of protein in the Ga extracts. A previous study reported that activated carbon could separate proteins in a mixture (Stone and Kozlov, 2014). However, these carbon materials are known to remove protein with larger molecules and retain the smaller peptides and amino acids. On the other hand, the method used for protein determination involved Coomassie staining. This method could have overestimated the total protein content in the Ga extracts.

#### Moisture and ash content

The moisture content of the lyophilized Ga extracts is shown in Table 2. The results showed that the BWC extract had the highest moisture content (21.14 %). All Ga stalk extracts, except for the BCS extract, had a moisture content lower than 10 %. The moisture content of all canopy extracts of Ga was significantly higher than the stalk extracts. Also, the moisture content of most B-type Ga extracts was higher than the R-type Ga extracts.

The high moisture content determined for these lyophilized Ga extracts could be due to their sugar components. The literature shows that sugar is susceptible to absorbing moisture in the environment (Jain and Roy, 2009). In this study, the high moisture content in the Ga canopy extracts could be due to their high sugar content. Other factors might contribute to the high moisture content of the Ga extracts, including humid weather, the duration of freeze-drying, and possible mishandling of samples. Although freeze-drying can remove free water molecules, some adsorbed moisture might be bound to sugar molecules in the extract. Therefore, oven-drying is more effective in removing water molecules adsorbed by the polysaccharides or sugar in the Ga extracts.

Ash analysis is another important measure for the Ga samples apart from their fat content (Table 2). All cultivated Ga samples had ash content of lower than 2 %. The results showed that the high ash content in the wild-type Ga might arise from a higher mineral content than the cultivated Ga, which could also be due to the higher polysaccharide content. The stalk samples of wild-type Ga also had a significantly higher ash content than their canopies (*P* < 0.05), but not for the cultivated Ga samples. Moreover, the ash content of the wild R-type Ga samples was significantly higher than the black samples (*P* < 0.01).

Total ash content is an important quality parameter for evaluating the nutritional composition of foods. Dry ashing has always been the typical procedure used to determine ash content in biomass. This ashing method is developed based on the principle that minerals are undestroyable by heat because they have lower volatility than the other components. Ga mushroom is a good source of essential minerals. It has an ash content of 1.8 %; potassium, phosphorus, chloride, and sulfur are the main mineral components besides carbon, oxygen, and hydrogen. The high ash content of the wild-type Ga could be due to the abundant minerals absorbed from the tree stump.

### Molecular weights and monosaccharide composition

As shown in Fig. S2, the corresponding molecular weights for the first peak ranging between 3057 and 3264 Da, whereas the second peak corresponded to the molecular weights ranged between 1393 and 1521 Da. The results showed that the canopy and stalk samples of wild B-type Ga (RWC and RWS) had the lowest molecular weight (1393.63 Da), whereas the stalk sample of cultivated R-type Ga (RCS) had the highest molecular weight (3263.75 Da).

A previous study reported that the molecular weights of polysaccharides extracted from *G. lucidum* using the ethanol precipitation method were higher than 10 kDa, and about 46 % of these polysaccharides had molecular weights of > 50 kDa (Liang et al., 2014). Hot water extraction breaks the glycosidic linkages based on high energy vibration, whereas amylase cleavages the glycosidic bonds. Therefore, the low molecular weights determined in the Ga extract could be due to the enzymatic reaction that markedly broke the glycosidic linkages of the polysaccharide.

Polysaccharide separation was performed based on size exclusion, where separating these polysaccharides in the Ga extract involved an exclusion mechanism. The stationary phase with a neutral membrane retains the neutral sugars based on their sizes (Harinarayan et al., 2006). Different molecular weights of polysaccharides are varied in their chain branches (Gaborieau and Castignolles, 2011). Separating substances with different molecular weights is also based on hydrophobic interaction and electrostatic force between the stationary phase and substances (Muhammad et al., 2022).

The Ga extracts could contain free monosaccharides, reducing sugars, and disaccharides in the water-soluble forms. As shown in Fig. S3, trehalose, mannose, glucose, galactose, xylose, and arabinose are detected in some Ga extracts, mainly in the RWC extract. Ribose was not detected in these Ga extracts. The saccharide ratios of these reducing sugars in the Ga extracts are shown in Table 2. All these saccharides were only determined in the RWC extract, and its ratio was 0.53:0.05:0.14:0.15:0.09:0.04 for trehalose, mannose, glucose, d-galactose, xylose, and arabinose, respectively.

Based on the disaccharide ratio, RWS and BWS extracts had the highest trehalose percentage. It was 97 % of the total saccharides content. The result shows that the stalks of wild-type Ga had a lower reducing sugar content than their canopy part. On the other hand, the cultivated R-type Ga extracts had the highest glucose proportion (7 %). The canopy extracts also had a higher glucose proportion than the stalk extracts, except for RCC and RCS. Moreover, mannose was only determined in BCC and RWC extracts but not in the other Ga extracts. Hence, mannose, galactose, xylose, and arabinose were solely detected in RWC.

The geographical origin of a Ga mushroom is an essential factor. Due to these Ga species being collected from two different geographical locations, environmental conditions and growing factors of these fungi were varied. Galactose, xylose, and arabinose were not detectable in most Ga extracts. They could be in trace. The extract purification using activated carbon could have removed some of these reducing sugars. The literature shows that a loss of oligosaccharides was noted during lignin removal using activated carbon (Hou et al., 2022). The possible adsorption mechanisms of sugar molecules by activated carbon were the attractive dispersion interactions between carbon surface and sugar molecules, competitive interactions among the hydroxyl group-containing solutes in the liquid, and non-idealities in carbon material and the extract solution phase (Chinn and King, 1999).

### Scanning electron micrograph and elemental analysis

**Fig. 1:**
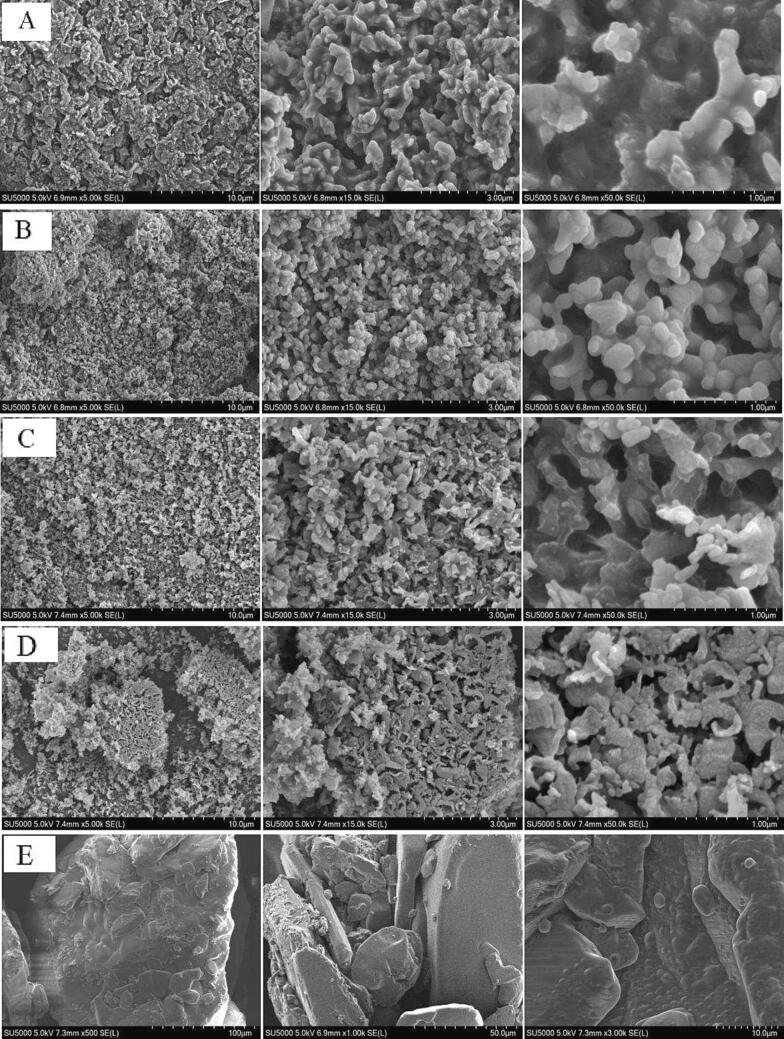

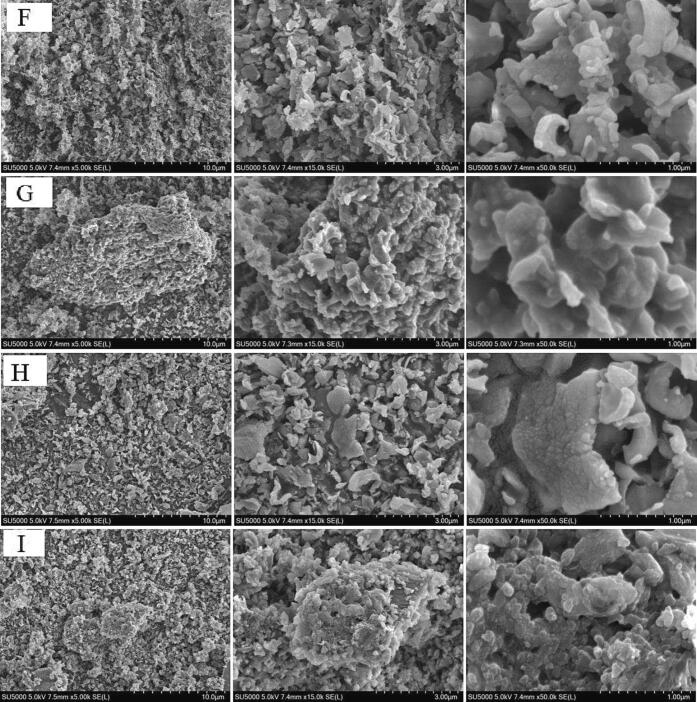
Scanning electron microscopic (SEM) images of R-type Ga extracts. (A) RCC, (B) RCS, (C) RWC, (D) RWS, (E) glucose, (F) BCC, (G) BCS, (H) BWC, and (I) BWS. The SEM images were taken with the magnifications of 5000, 15000, and 50000 × for the Ga samples; the images were taken with the magnifications of 500, 1000, and 3000 × for glucose standard.

SEM images of the enzyme-treated Ga extracts are depicted in Fig. 1. The SEM images were microstructures of these freeze-dried extracts. The SEM images showed that the enzyme-treated Ga samples had a multiporous particle surface compared with the glucose particles. The surface view of the glucose particles at a magnification of 500 × showed that the particle surface was smooth and without notable pores. Therefore, the commercial glucose powder is not a porous material. The particle surface view of the Ga extracts was multiporous, where the canopy extracts of Ga samples had higher percentages of macropores of more than 10 μm than the stalk extracts. The Ga canopy extracts also had macropores between 1 and 2 μm lesser than their stalk extracts, although it was not visible in the SEM images. The SEM images showed that the particles of Ga stalk extracts had pore sizes smaller than the canopy extracts.

As shown in Fig. S4, the particle of non-enzyme-treated RCC extract is fibrous and ovoid forms. Limited macropores between 1 and 2 μm were noted for this extract. The SEM images showed that the enzymatic treatment remarkably altered the structure of these Ga extracts. The SEM images also revealed the significance of studying different parts and types of Ga. The particles of canopy and stalk extracts of these Ga species had minor differences in pore sizes, roughness, particle shape, and other morphological characteristics.

The surface morphology of polysaccharide particles was affected by using different sample preparation methods. The porous structure of a polysaccharide could be the gap from glycosidic linkages between two glucose molecules. Freeze-drying of the polysaccharide-containing extract caused the formation of highly porous polysaccharide structures. Therefore, there is a need to analyze the microstructure of glucose. As shown in Fig. 1E, the surface morphology of glucose powder is not porous. It could be because glucose had not been lyophilized using a freeze dryer. The literature showed that the particle surface of the ultrasonic-treated spores of *G. lucidum* had granular fractures, structural disintegration, and other morphological changes (Zhao et al., 2014). The particles of the non-enzyme-treated extracts had a larger lamellar structure even after ultrasonication. The molecular aggregation of these polysaccharide molecules could be closely related to the presence of carboxyl and hydroxyl groups (Ji et al., 2021).

In combination with EDS, SEM was used to examine the spatial distribution of the emitted backscatter secondary electrons from the extract granule (Layton-Matthews and McClenaghan, 2021). The energy spectra of these secondary electrons emitted are corresponded to specific elements. The EDS spectra reflect the differences in the average atomic numbers of an area of the extract granule. The Ga extracts were also determined for their main bulk elements, macrominerals, microminerals, and heavy metals (Table 3). Among the four main bulk elements, oxygen was most abundant in the extracts, followed by carbon, sulfur, and nitrogen. The stalk extracts had a higher oxygen percentage than the canopy extracts, except for the RWS extract. RCC extract had the highest carbon content. Nitrogen was not detected in RCS. The wild-type RWC and RWS extracts contained nitrogen, but nitrogen was not detected in the wild-type black Ga extracts. The other 0.02 % was composed of macrominerals like calcium and sodium.

**Table 3:** Elemental composition on the surface of purified enzyme extracts of different Ga samples.

**Element [%]**	**Glu**	**RCC**	**RCS**	**RWC**	**RWS**	**BCC**	**BCS**	**BWC**	**BWS**
***Bulk elements***									
**C**	56.94	39.93	7.31	14.74	15.31	23.12	12.58	12.82	8.39
**O**	42.87	51.73	68.41	63.93	63.86	50.79	68.73	64.01	70.65
**N**	–	0.48	–	2.22	2.85	2.39	–	–	–
**S**	–	7.03	16.30	12.57	9.75	6.19	10.37	12.05	11.70
***Macrominerals***									
**Ca**	0.01	0.01	0.01	0.15	0.15	0.31	0.23	0.02	1.01
**Cl**	–	–	1.08	0.34	0.50	0.49	0.34	1.12	0.37
**P**	–	0.07	3.78	3.28	4.26	4.86	2.99	6.81	3.46
**K**	–	0.14	1.41	1.68	1.48	9.90	2.78	1.07	3.32
**Na**	0.01	–	–	–	–	–	–	–	–
***Microminerals***									
**Fe**	–	0.01	0.01	0.04	0.01	0.01	0.04	0.07	0.03
**Cu**	–	0.01	0.02	0.04	0.04	0.06	0.03	0.03	0.02
**Zn**	–	–	–	–	–	–	0.04	–	–
**Ge**	–	0.22	0.25	0.28	0.30	0.15	0.34	0.22	0.28
***Heavy metals***									
**As**	–	0.01	0.16	0.13	0.18	0.24	0.30	0.05	0.13
**Hg**	–	–	–	–	–	–	–	–	–
**Cd**	–	–	0.01	0.01	0.01	0.07	0.02	–	0.02
**Ni**	–	–	0.01	0.02	0.02	0.01	0.04	–	–
**Zr**	–	–	1.23	–	1.22	1.19	1.10	1.70	–
**Sn**	–	–	0.02	0.06	0.04	0.23	0.07	0.03	0.08
**Pt**	0.18	0.36	–	0.52	–	–	–	–	0.54

All Ga extracts contained calcium, phosphorus, and potassium as their macrominerals. Sodium was not detected in these extracts, whereas chloride was not found in the RCC extracts. The percentages of phosphorus and potassium were even higher than the nitrogen percentage in the BCC extract. It also contained the highest level of micromineral, such as copper, and the lowest germanium percentage among the Ga extracts. Zinc was the only micromineral detected in the BCS extract. It also had the highest germanium percentage. Moreover, the BWC extract had the highest iron content. All stalk extracts of Ga had a germanium level higher than the canopy extracts.

Heavy metals are the toxic elements that affect human health. The data obtained from the EDS analysis showed that the stalk extracts of all B-type Ga had percentages of heavy metals lower than their canopy extracts, but not for the other two Ga species. RCC extract was the least toxic because it only contained 0.01 % arsenic. Other heavy metals were not found in the RCC extracts. Similarly, some heavy metals were not detectable in RWC and BWC extracts (Table 3). Although the BCC extract had high percentages of the essential elements, it also contained high arsenic, cadmium, zirconia, and stanum levels. On the other hand, mercury was undetected in all Ga extracts. The detected amount of platinum could arise from the coating material for SEM analysis.

### Determination of functional groups by FTIR

**Fig. 2:**
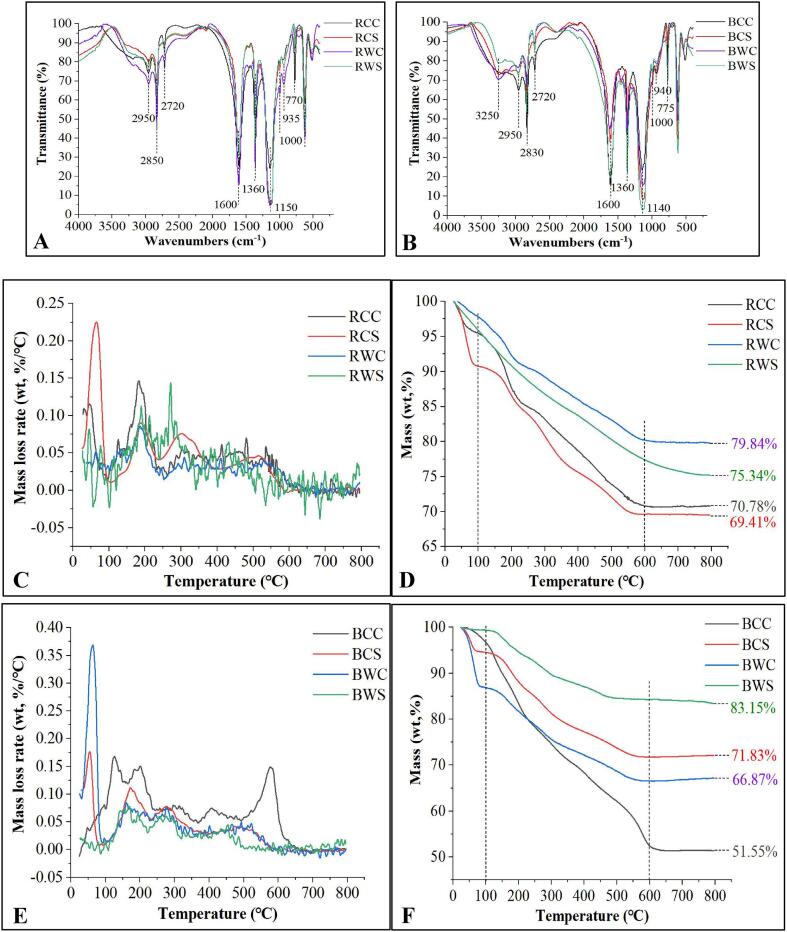
Infrared spectra of red (A) and black (B) Ga extracts at wavenumbers ranging 4000–400 cm^−1^, and thermal decomposition patterns of (C and D) red and black (E and F) Ga samples treated with temperatures from 30 to 800 °C. (For interpretation of the references to color in this figure legend, the reader is referred to the web version of this article.)

The FTIR spectra of the enzyme-treated Ga extracts are shown in Fig. 2. The spectrum peaks of these Ga extracts were mainly distributed in the spectral regions ranging between 3300 and 2700 cm^−1^, between 1660 and 900 cm^−1^, and between 800 and 500 cm^−1^. The peaks in the spectral region of over 3000 cm^−1^ belonged to the —OH band of sugar and saponin structures of the Ga extracts (Wang et al., 2011), whereas the IR peaks at 2950–2720 cm^−1^ spectral region were the stretching vibration of the C—H bonds on the saturated carbon (Sun et al., 2008). The IR peak at 2720 cm^−1^ was the multiple stretching vibrations of —CH, whereas the IR peak at 2950 cm^−1^ was the multiple stretching vibrations of = CH. Besides these, ≡CH (∼3200 cm^−1^) was not found in the Ga extract. The IR peak at 2850 cm^−1^ was mainly the stretching vibration of −OH from a carboxylic acid. The absorption peaks of hydroxyl groups of these reducing sugars were not detectable, where these hydroxyl groups should have moderately strong stretching vibrations at the spectral region of > 3500 cm^−1^.

The intense absorption spectrum near the 1600 cm^−1^ region was due to either vinyl or aromatic C=C bonding. However, the IR spectrum at 1600 cm^−1^ of BCS could be due to the C=N bond. The N—H bonding of the Ga extracts was found at the IR spectrum of 1600 ± 50 cm^−1^ region. This N—H bond showed the existence of an amino group. The absorption peaks between 1920 and 1620 cm^−1^ spectral region were the C=O bonding of the different compound structures. The IR peaks in the 1400–1300 cm^−1^ spectral region were the bonding vibration of C—H, which were also the characteristic absorption of the infrared spectrum of the compounds in the Ga extracts. The intense absorption spectrum at 1360 cm^−1^ indicated the presence of a CH_3_ bond. The weak absorption spectra at 1200–1000 cm^−1^ region showed the presence of C—O—C bonding. The strong absorption spectra near the 1150 cm^−1^ region mainly indicated the presence of either C—O or C—C bonding (Chen et al., 2023), which might be the stretching vibration of the pyranose ring.

The FTIR data showed that the polyglycosidic bond and polysaccharide conformation among the Ga extracts were similar. The polysaccharides and saponins extracted from these two parts of Ga fruiting bodies had comparable molecular structures. The literature shows that the IR peaks in the 1130–980 cm^−1^ region represented the polysaccharides of *G. lucidum* (Chen et al., 2012). The characteristic peak at 1240 cm^−1^ might indicate the presence or absence of sulfate (Melo et al., 2002). The absorption peak of the Ga extracts at 1240 cm^−1^ was weak. Therefore, C=S was not the key functional group in the Ga extracts.

The result obtained from this study showed that the IR peak at 1600 cm^−1^ was C=C bonding. However, a previous study reported that the intense absorption spectrum near the 1600 cm^−1^ region was due to the C=O group (Ge et al., 2009). The C=O bond found for the Ga extracts had absorption spectra between 1920 and 1620 cm^−1^ spectral region. The Ga extracts could contain other nitrogen-containing compounds, such as purine and pyrimidine, besides the amino acids due to the C=N bond found in the BCS extract. The result also indicated that ultrasonication did not affect the main functional groups of compounds.

### Pyrolysis characteristics

The thermal conductivity and thermal gravity of the Ga extracts are depicted in Fig. 2. Based on the thermal conductivity analysis, the patterns of mass decomposition for the Ga extracts were varied. As the energy increased during the drying stage, the compounds dehydrated until they reached a maximum dehydration rate at 65 ± 2 °C. The initial weight loss occurred at between 40 and 100 °C. Applying a heating rate of 10 °C/min, the moisture in the extracts completely evaporated, leading to the first heat loss.

In stage two, the weight loss occurred between 100 and 600 °C. It was a devolatilizing process. The decomposition processes involved a few sub-stages for some Ga extracts, followed by carbonization. The occurrence of a few sub-stages of material decomposition could be because these Ga extracts contained a range of nutritional components, such as reducing sugars, short-chain polysaccharides, amino acids or some short-chain peptides, and even saponins. The decomposition and depolymerization of these components in the Ga extracts resulted in the second stage of weight loss when the temperature continued to rise (Yang et al., 2017). When the heating temperature increased from 600 °C to 800 °C, the weight of the extracts remained constant. It could be due to most components decomposed and carbonized at this stage. The third phase was the char oxidation stage (Kumar et al., 2021).

Based on the results obtained from this study, BCC had the highest weight loss rate, whereas BWS had the least. It indicated that the nutritional components in BWS had the best thermal stability. The weight loss at the initial stage might be due to the phase transition, whereas the weight loss in the second stage could be due to decomposition (Prajapati et al., 2013). The weight loss rate remained constant because most organic components decomposed (Mishra and Pal, 2007). The BCC extract had poor thermal stability because it had high sugar content. This ezyme-treated extract contained the most sugar components compared with the other Ga extracts. On the contrary, BWS had the lowest thermal stability because it constituted the least TSC.

The literature shows that the percentages of neutral sugar in Ga polysaccharides obtained using both hot (60 °C) and cold (−30 °C) extraction methods were similar (Fan et al., 2012). Another study reported that the polysaccharides extracted even with the hot compression method had a high thermal stability at temperatures > 100 °C. These findings revealed that the polysaccharides in Ga mushroom were heat stable. The information on the thermal stability of Ga polysaccharides is necessary for using these components as drugs and cosmetic materials as the material processing commonly involves high-temperature treatment.

### Emulsification activity

**Fig. 3:**
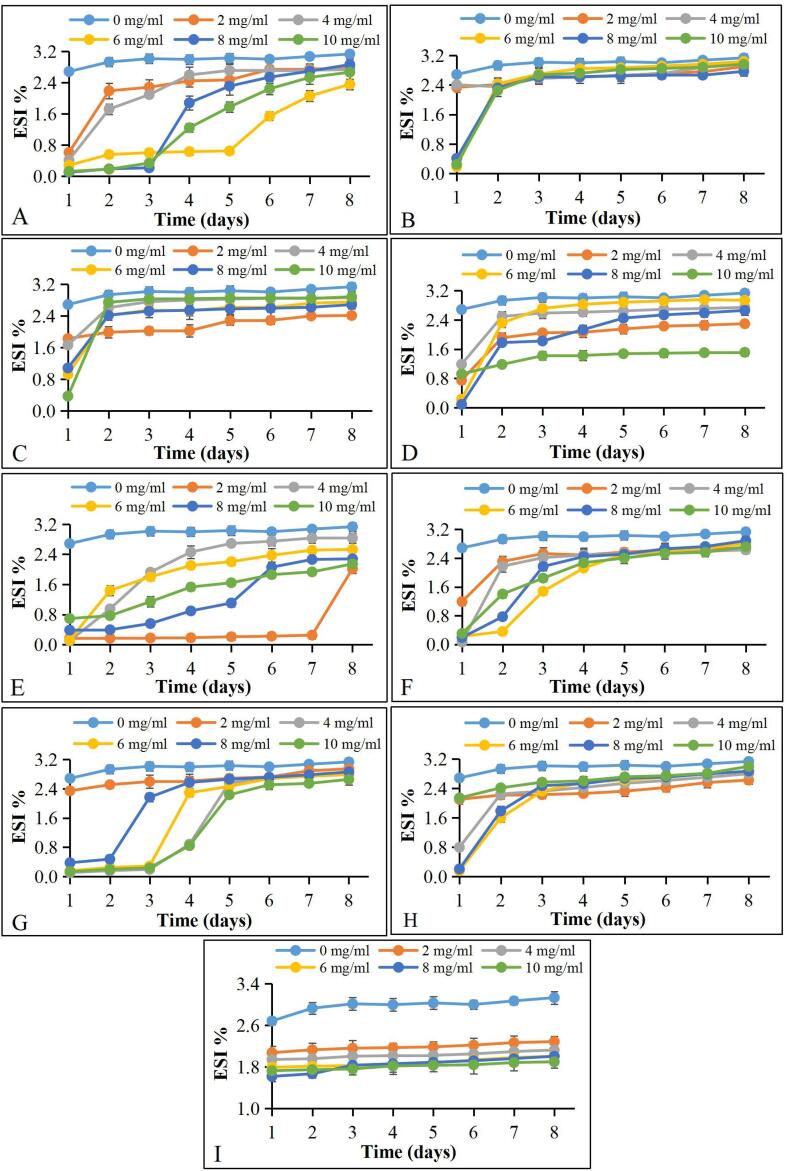
Emulsion separation index (ESI) of Ga samples (A) RCC, (B) RCS, (C) RWC, (D) RWS, (E) BCC, (F) BCS, (G) BWC, (H) BWS, and (I) lecithin.

The emulsion separation index (ESI) values (%) of these Ga extracts are shown in Fig. 3. The results showed that the ESI values were significantly different between B-type and R-type Ga extracts (*P* < 0.05). The Ga extracts at different extract concentrations also showed different emulsion separation patterns (Fig. S5) during the eight days of storage at a room temperature of 25 °C. The ESI values of different concentrations of all Ga extracts were significantly lower than the control (2.69 ± 0.07 %). The results showed that these Ga extracts have emulsifying activity. The statistical results showed that the Ga extracts had an emulsification effect comparable to lecithin. At the initial stage, the ESI value of lecithin was higher than some of the Ga extracts. The lecithin-based emulsion also remained stable even up to eight days. The ESI (%) of lecithin was also concentration-dependent, but not for the Ga extracts.

A low ESI (%) of these extracts could be due to their high saponin content. Although the BCC extract had the highest total saponin content, its ESI value on day 8 remarkably increased. The ESI values of this extract at 2 mg/mL remained low until day 7. Moreover, the ESI values of RWS at an extract concentration of 10 mg/mL were constantly lower (1.5 ± 0.03 %) from day 5 to day 8. It showed that the Ga saponin could stabilize the emulsion for about a week.

The literature shows the use of different concentrations of lecithin as an emulsifier to reduce the interfacial tension of an emulsion besides improving its emulsion stability (Sui et al., 2017). Adding these Ga extracts and lecithin to a mixture of oil and water for emulsification should produce a creamy layer. However, the emulsion prepared by adding lecithin did not show the expected foam layer. We also noted that these Ga extracts had better foaming ability than the natural soy lecithin. The emulsification activity of these Ga extracts was attributed to the high Ga saponin content. Due to saponin contains hydrophobic triterpenes and hydrophilic sugar molecules, it is an effective emulsifier. The variation in ESI values found in the Ga extracts could be due to these extracts containing short-chain polysaccharides, monosaccharides, and amino acids besides saponin.

The emulsification activity of plant polysaccharides can be increased further by adding natural emulsifiers. An emulsifier should possess the ability to accumulate and stabilize oil and water in an emulsion. Studying a novel natural emulsifier isolated from Ga mushroom to replace surfactants in the cosmetic industry is indispensable because synthetic surfactants added to cosmetic products irritate the skin (Mijaljica et al., 2022). Hence, studies on natural emulsifiers isolated from plants have become a new trend. These plant-based emulsifiers can be used in the food and cosmetics industries to replace synthetic emulsifiers and surfactants.

### In vitro antioxidant activity

As shown in Table 2, the RWS extract has the lowest EC_50_ value. The EC_50_ values of the other Ga extracts were higher than 15 mg/mL. No significant difference in EC_50_ values between RCC and BCS (*P* > 0.05). The B-type Ga extracts had lower DPPH radical scavenging activities than the R-type Ga extracts, especially the wild B-type Ga extracts. Also, the wild R-type Ga extracts had higher DPPH radical scavenging activities than those cultivated Ga extracts. On the other hand, RCS, RWS, and BCS extracts had the highest FRAP values (Table 2), whereas the FRAP values of RCC, RWC, and BCC extracts were significantly lower than their stalk extracts (*P* < 0.05). Both canopy and stalk extracts of wild B-type Ga were the remarkably lowest (*P* < 0.01).

The results showed that RWS extract had the best antioxidant activities compared to the other Ga extracts. It is because this Ga extract had the lowest and highest EC_50_ and FRAP values, respectively. Although RCC, RCS, and BCS extracts had a high reducing ability, their DPPH radical scavenging activities were not higher than the other Ga extracts with lower FRAP values. It shows that the DPPH radical scavenging of these Ga extracts was not correlated with the reducing capacity assessed using FRAP assay. DPPH radical scavenging activity of the BCC extract was low because it had a high sugar and saponin content. The high DPPH radical scavenging activity of wild R-type Ga extracts could be due to these extract were obtained from the wild-type Ga samples. The free radical scavenging activities of these extracts involve transferring an electron from the oxygen molecules of the hydroxyl group to the reactive species with unpaired electrons or hydrogen. The antioxidant activities determined for the Ga extracts showed they are potential emulsifiers with antioxidant capacity for cosmetic application.

## Conclusion

The study of the canopy and stalk of different Ga species revealed the effectiveness of enzyme digestion combined with ultrasonic-assisted hot-water extraction. This technique remarkably increased the extraction yield, total sugars, and total saponin content compared with the ultrasonic-assisted hot-water extraction without enzyme treatment, especially for the cultivated black Ga sample (BCC). It is a sustainable extraction method for isolating polysaccharides and saponins from tubers and roots with high extraction yields. The extraction yield and TSC of the enzyme-treated BCC increased over three times higher than the sample without the enzyme treatment, whereas its total saponin content was about two times higher than the non-enzyme-treated sample.

This study is fundamental research on the characterization of polysaccharides and saponins extracted from the Ga samples. Adding amylase to the extraction medium enhanced the extraction of polysaccharides and saponins in the Ga samples. These extracts are potent sources of emulsifiers for food processing and cosmetic formulation. Applied experimental research has not been performed. It is the main limitation of this study. A comparative study of saponin in these wild-type and cultivated Ga mushrooms in preventing diseases and structural modification of the Ga saponin with acid-base or hydrocarbon chains are suggested for future studies to make it a pharmaceutical ingredient and surfactant in cosmetic formulas.

## Declaration of competing interest

Hock Eng Khoo and Xia Li are the principal investigators of these research funds.

## CRediT authorship contribution statement

**Bo Jie Chen:** Investigation, Methodology, Writing – original draft. **Yang Liu:** Methodology, Investigation. **Ke Yang:** Methodology, Formal analysis. **Xia Li:** Funding acquisition, Software, Supervision. **Xinhong Dong:** Data curation, Writing – review & editing. **Yuan Guan:** Validation, Supervision. **Amin Ismail:** Conceptualization, Resources. **Hock Eng Khoo:** Project administration, Funding acquisition, Supervision, Writing – review & editing.

## Declaration of Competing Interest

The authors declare the following financial interests/personal relationships which may be considered as potential competing interests: Hock Eng Khoo reports financial support and equipment, drugs, or supplies were provided by Guilin University of Technology. Hock Eng Khoo reports equipment, drugs, or supplies and travel were provided by Science and Technology Department of Guangxi Zhuang Autonomous Region.

## Data Availability

Data will be made available on request.
