# Supplementary material for: Amylase-assisted extraction alters nutritional and physicochemical properties of polysaccharides and saponins isolated from Ganoderma spp
Source: Food Chem X. 2023 Sep 29;20:100913. doi: 10.1016/j.fochx.2023.100913 (PMC10740064; doi:10.1016/j.fochx.2023.100913)
Supplement: Supplementary data 1 [file mmc1.pdf]

Supplementary Figures

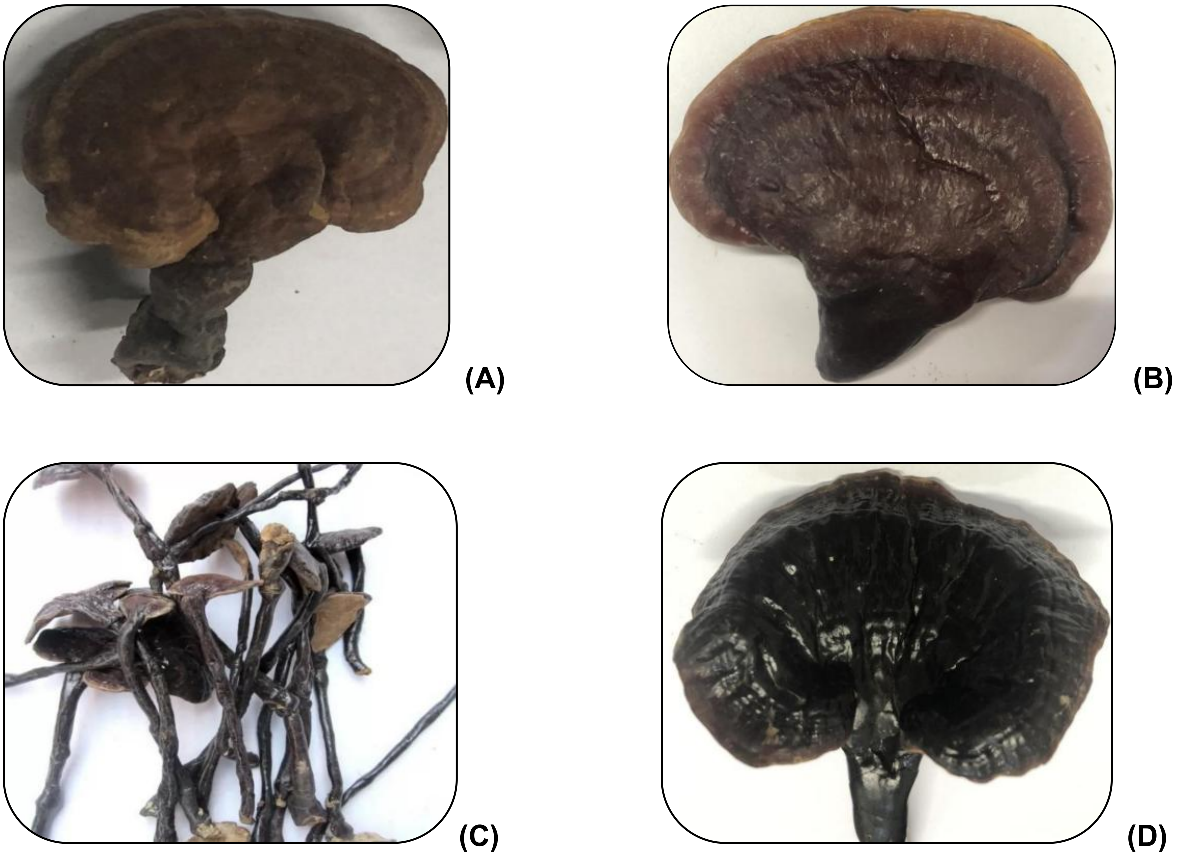

Fig. S1. Images of *Ganoderma* samples. (**A**) Wild-type brownish *G. applanatum*, (**B**) cultivated purplish *G. sinensis*, (**C**) wild-type black *G. hainanense*, and (D) cultivated *G. atrum*.

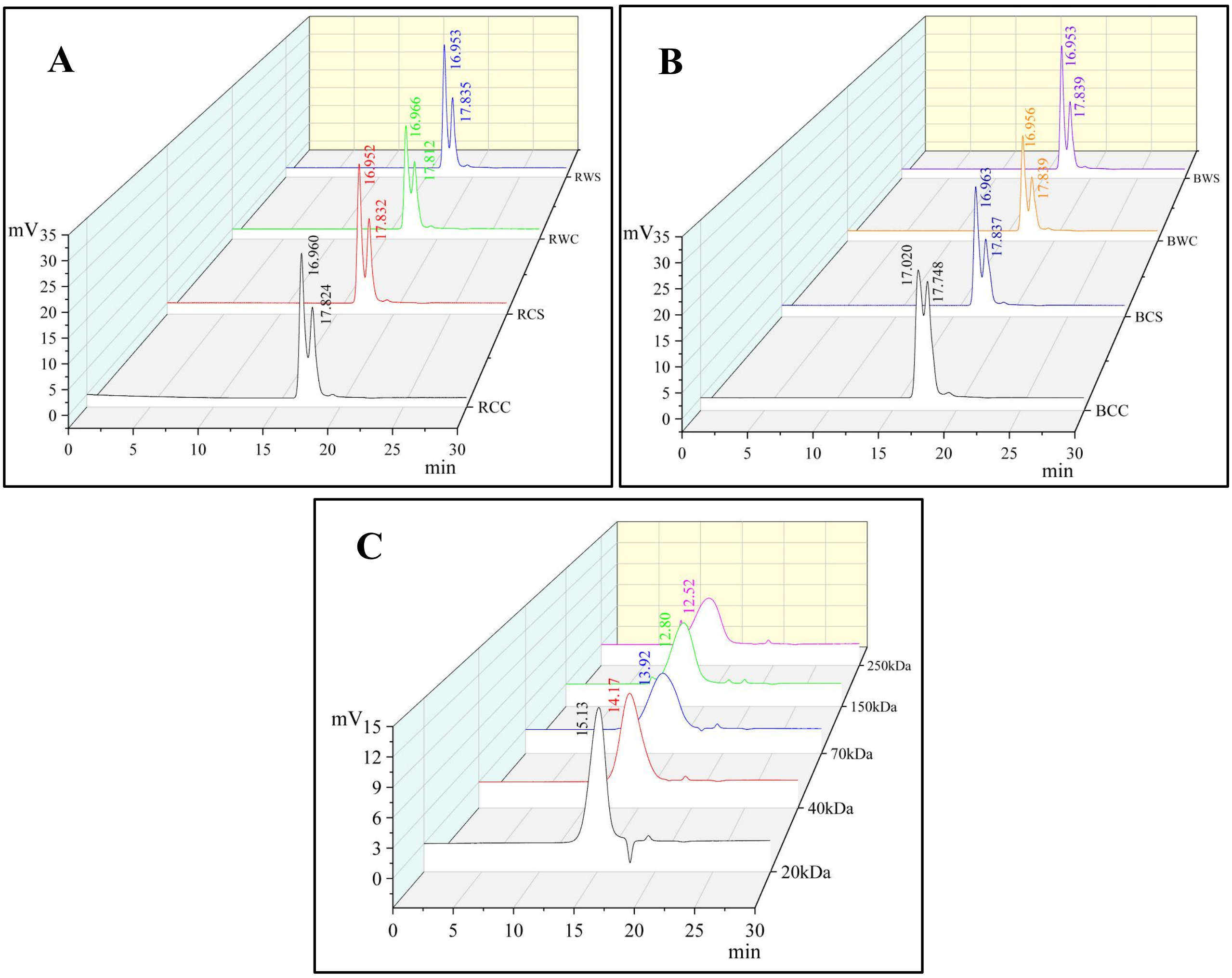

Fig. S2. HPLC chromatograms of different molecular weights of the *Ganoderma* (Ga) extracts. (**A**) R-type Ga extracts, (**B**) B-type Ga extracts, and (**C**) different molecular weights of dextran standard. *RCC, canopy of the R-type Ga cultivated extract; RCS, stalk of the R-type Ga cultivated extract; RWC, canopy of the R-type wild Ga extract; RWS, stalk of the R-type wild Ga extract; BCC, canopy of B-type cultivated Ga extract; BCS, stalks of B-type cultivated Ga extract; BWC, canopy of B-type wild Ga extract; BWS, stalk of B-type wild Ga extract.

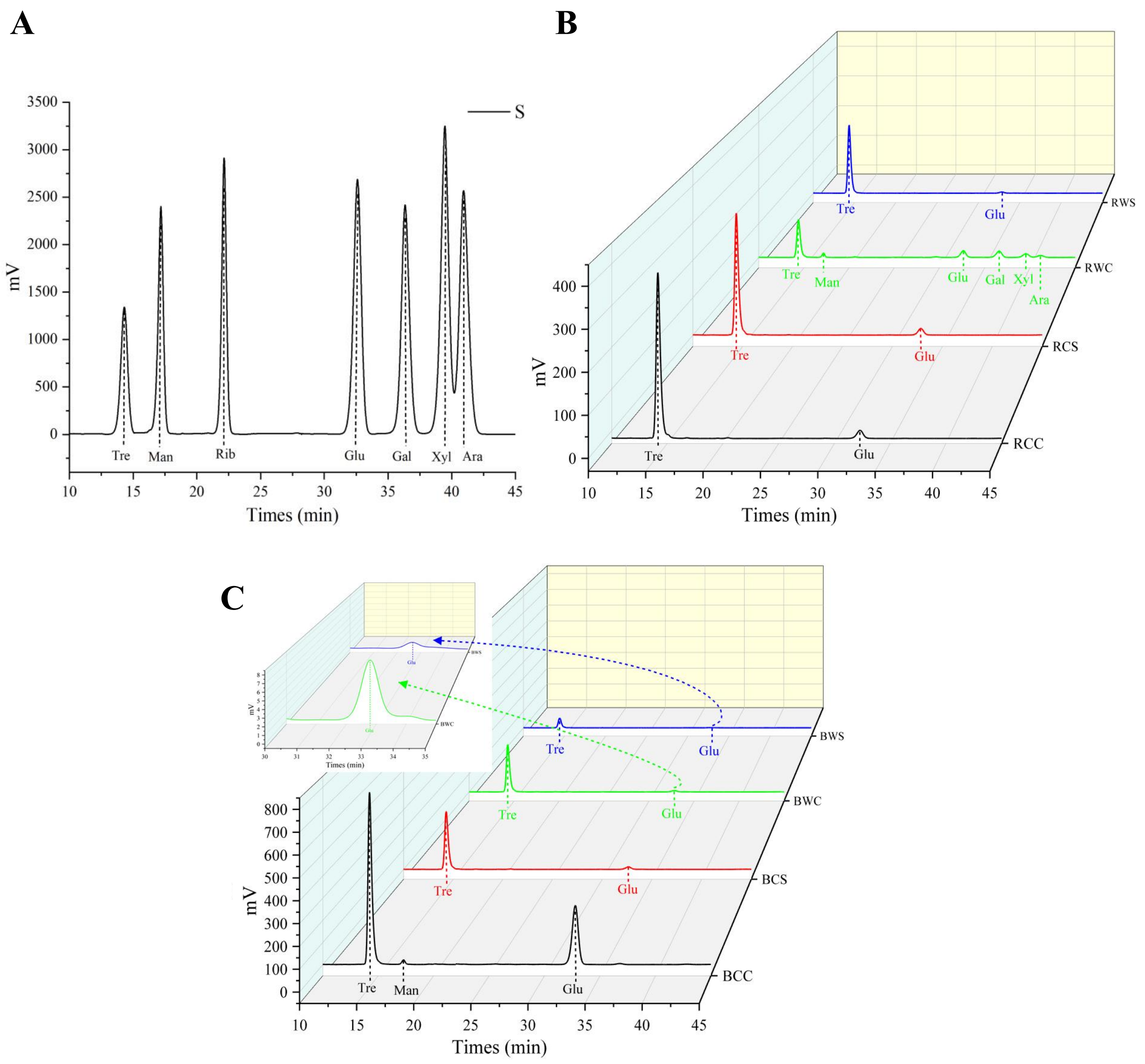

Fig. S3. HPLC chromatograms of different monosaccharides in the *Ganoderma* (Ga) extracts. Elution times of the monosaccharide peaks for (**A**) sugar standards, (**B**) R-type Ga extracts, and (**C**) B-type Ga extracts. *RCC, canopy of the R-type Ga cultivated extract; RCS, stalk of the R-type Ga cultivated extract; RWC, canopy of the R-type wild Ga extract; RWS, stalk of the R-type wild Ga extract; BCC, canopy of B-type cultivated Ga extract; BCS, stalks of B-type cultivated Ga extract; BWC, canopy of B-type wild Ga extract; BWS, stalk of B-type wild Ga extract.

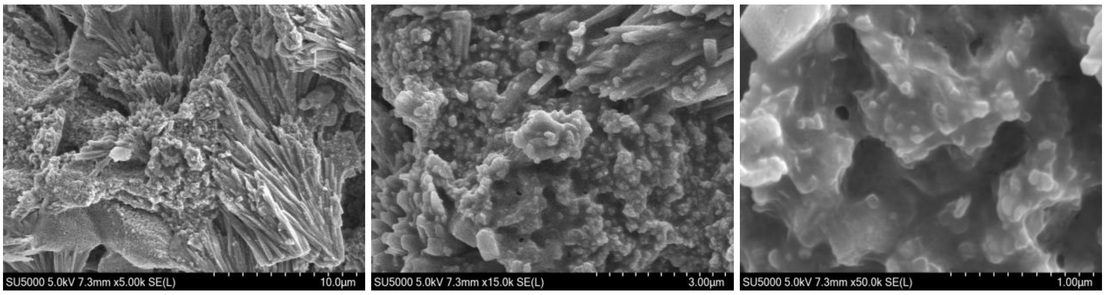

Fig. S4. Scanning electron microscopic (SEM) images of the canopy extract of R-type cultivated Ga. The SEM images were taken with the magnifications at 5,000, 15,000, and 50,000.

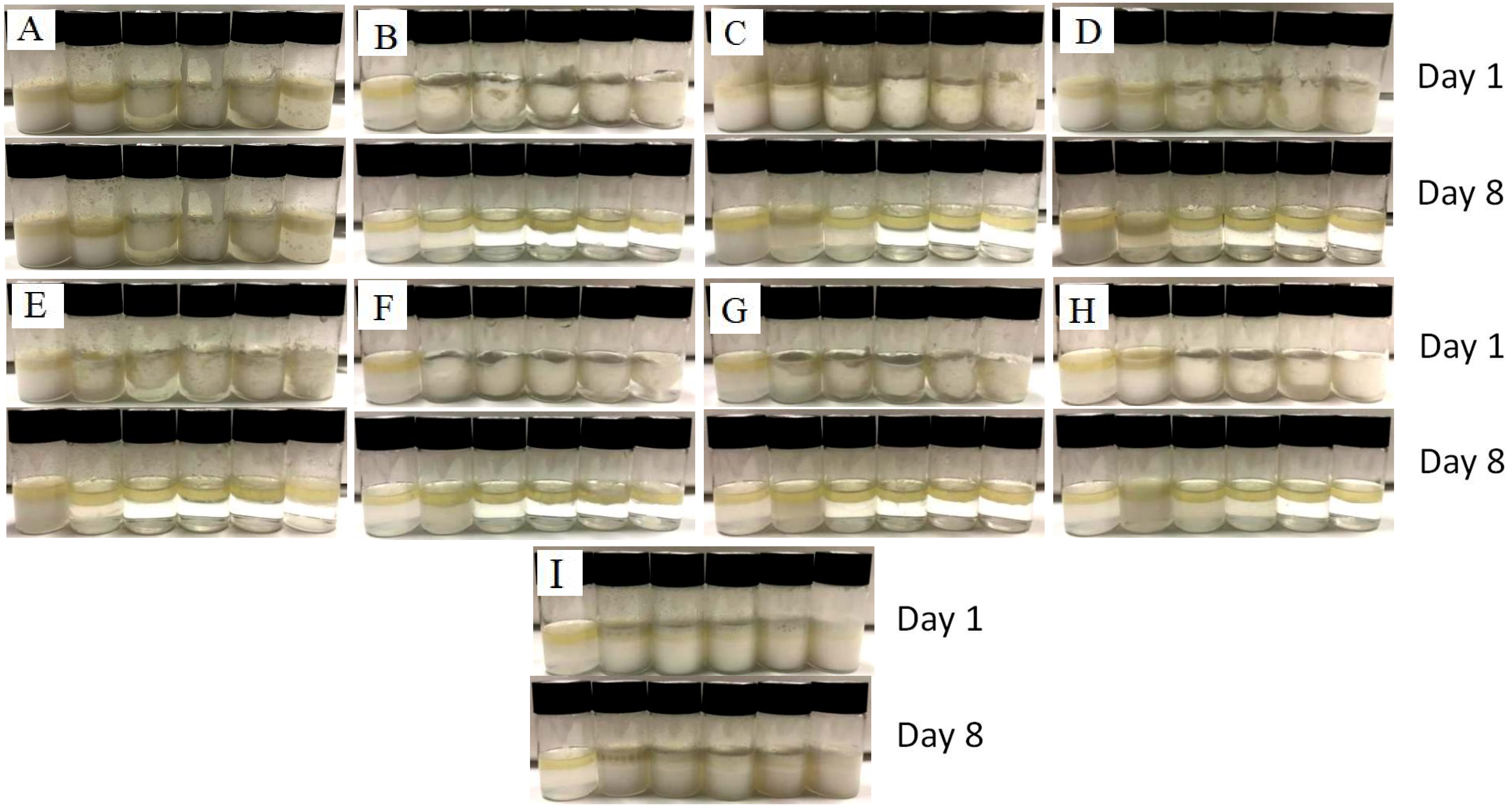

Fig. S5. Emulsion layers of the Ga extracts (**A**) RCC, (**B**) RCS, (**C**) RWC, (**D**) RWS, (**E**) BCC, (**F**) BCS, (**G**) BWC, (**H**) BWS, and (**I**) lecithin. *The sample and standard concentrations from left to right are 0, 2, 4, 6, 8, and 10 mg/mL, respectively.
